# Barriers and facilitators to patient recruitment to a cluster randomized controlled trial in primary care: lessons for future trials

**DOI:** 10.1186/s12874-015-0012-3

**Published:** 2015-03-12

**Authors:** Juliet M Foster, Susan M Sawyer, Lorraine Smith, Helen K Reddel, Tim Usherwood

**Affiliations:** Clinical Management Group, Woolcock Institute of Medical Research, University of Sydney, Sydney, Australia; Centre for Adolescent Health, Royal Children’s Hospital Melbourne, Melbourne, Australia; Department of Paediatrics, The University of Melbourne, Melbourne, Australia; Murdoch Childrens Research Institute, Melbourne, Australia; Faculty of Pharmacy, University of Sydney, Sydney, Australia; Department of General Practice Sydney Medical School Westmead, University of Sydney, Sydney, Australia

**Keywords:** Attitude of health personnel, Patient selection, General practitioners, Physician-patient relations, Randomized controlled trials as topic, Asthma/prevention & control

## Abstract

**Background:**

Primary-care based randomized controlled trials (RCTs) build an important evidence base for general practice but little evidence exists about barriers to recruitment which often hamper such trials.

We investigated the issues that impeded and facilitated recruitment to a clinical trial in general practice.

**Methods:**

GPs participating in a cluster RCT that tested interventions for improving medication adherence and asthma control completed a survey comprising quantitative and free text questions about their recruitment experiences. We used backward regression to analyze quantitative data and coded free text responses into themes.

**Results:**

40/55 of enrolled GPs recruited patients, but only one-third reached the planned recruitment target (5 patients/GP). In univariate analyses, poor patient recruitment by GPs was significantly associated with longer time to first patient enrolment, GP-perceived poor access to eligible patients and GP working in a practice training medical students. In regression analysis, only the first was significant (p = 0.001); the explained variance of the model was 48%. Themes from free text responses described recruitment barriers at the level of GP (e.g. GPs excluding patients for whom research appeared too challenging), practice (e.g. practice cultures disempowered GPs), patient (e.g. reluctance to change treatment for research) and study (e.g. protocol requirements complicating recruitment). Facilitators included GPs perceiving good support from the research team.

**Conclusion:**

Targeted recruitment support early in the recruitment phase may enhance recruitment rates. Over time, interventions to enhance a general practice research culture are also likely to enhance skills to recruit patients, even for complex interventions. We recommend systematic evaluation of recruitment approaches and outcomes in future RCTs to optimize feasibility and success of these important trials.

**Trial registration:**

Australian and New Zealand Clinical Trials Registry ACTRN12610000854033 (date registered 14/10/2010).

## Background

Primary-care based randomized controlled trials (RCTs) are important for building an evidence base relevant to general practice in which most clinical decisions are made [1]. Such trials are particularly relevant in conditions treated principally by general practitioners (GPs), such as asthma [2]. However, poor recruitment of research participants by GPs reduces the efficiency of studies due to extended recruitment time, the need to enroll greater numbers of GPs than planned and failure to reach patient recruitment targets, which are each factors that threaten the ethics of the research if the result has insufficient statistical power [3]. As with any research, better understanding of the facilitators and barriers to recruitment underpins the success of future trials [4].

A recent systematic review of recruitment strategies for primary care research highlights the paucity of current research evidence and has called for researchers to include more evaluation of recruitment strategy in trials [5]. Existing research on patient recruitment by GPs describes a range of potential enablers and facilitators [6-14], but tends to rely on narrative reviews or inferences based on researchers’ rather than GPs’ experiences [15], providing little insight into which issues are more or less important, or how recruitment barriers differ in different types of RCTs, particularly from the perspective of GPs recruiting for these studies.

One important subset of primary care research consists of “professional-cluster” RCTs, which typically require GPs to receive training in a specific intervention, such as counseling, which they subsequently deliver to patients in their own practice [16]. Such studies can provide useful data about the effectiveness of interventions in primary care but may create novel recruitment problems given the high level of research responsibility often required of participating GPs [17].

In a primary care-based professional-cluster RCT of interventions to improve adherence and disease control in adults with asthma (the Management to Improve Control of Asthma or ‘MICA’ study) [18], we aimed to investigate the barriers and facilitators to patient recruitment as perceived by GPs responsible for patient recruitment.

## Methods

### MICA professional-cluster RCT study design

The MICA study was funded by the Australian National Health and Medical Research Council (NHMRC) to measure the effectiveness of two different primary care interventions to improve asthma control and medication adherence in adults with poorly controlled asthma. The study design and methods are described in detail elsewhere [18].

In summary, GPs were randomly allocated in a 2×2 factorial design to active or control group for each of two interventions, giving four intervention groups: (1) Active Usual Care (UC); (2) Inhaler Reminders and Adherence Feedback (IRF); (3) Personalized Adherence Discussions (PAD); or (4) IRF + PAD. GPs in all groups provided Active Usual Care (including a written asthma action plan and an inhaler technique check) and provided one month’s supply of a suitable controller inhaler, a digital peak flow meter, and a spacer if needed.

The study interventions are outlined below.

1. Inhaler Reminders and Adherence Feedback (IRF): Reminders for missed doses of controller therapy were delivered by an electronic device fitted to the patient’s inhaler [19]. Objectively measured medication use data were automatically uploaded to a secure website, which patients and their own GP could view and discuss.
2. Personalized Adherence Discussions (PAD): GPs were trained to utilize brief motivational interviewing and collaborative goal-setting techniques to discuss each patient’s personal medication/disease beliefs and concerns.

All participants were required to use a pressurized metered dose inhaler (the most common device for controller medication in Australia) to allow adherence monitoring and data upload. Patients not using this type of inhaler for their controller medication were prescribed it at the same/equivalent dose by their GP at study entry.

All GPs attended one education workshop prior to enrolling patients. The workshop outlined current best-practice in the diagnosis and treatment of asthma and reviewed the evidence for the study interventions prior to practical skills training (this was considered key to GP engagement and confidence in delivering the interventions [13]). GPs received intervention support tools to minimize GP burden; these had been previously piloted with non-trial GPs. GPs could commence patient recruitment immediately upon completing their workshop. Throughout the study each GP was contacted fortnightly either by telephone, fax or newsletter. At least once per month GPs were offered top-up education/support via telephone by a workshop facilitator who represented the research team.

### MICA RCT: recruitment of GPs

GPs received a personally addressed invitation fax or letter, co-signed by a local Professor of General Practice, a Respiratory Physician and the Director of the local general practice regional organization; a study information sheet and expression of interest fax form were enclosed. GPs received one follow-up telephone call to confirm receipt of the invitation and allow GPs to ask questions about the study. GPs were allocated continuing professional development points (a requirement for continuing registration as a GP) for attending a training workshop and additional points for trial participation. GPs were reimbursed $100 for each patient enrolled. The original target number of GPs was 44.

### MICA RCT: inclusion criteria for GPs and patients

GP inclusion criteria were: access to a computer and email, and not currently participating in any other adherence-promoting study. To minimize cross-contamination between groups, only one GP was enrolled per practice.

Key patient inclusion criteria were: 14–65 years of age, prescribed twice daily inhaled corticosteroid/long-acting β_2_-agonist for ≥1 month and sub-optimal asthma control (Asthma Control Test (ACT) ≤19) [20] but without asthma exacerbations in the last month. We planned for recruitment of 220 patients from 44 practices, with each GP enrolling 5 patients within a target 6 month period.

### MICA RCT: GP patient-recruitment strategies

GPs invited patients from their own practice to participate. GPs were provided with a waiting room advertisement poster and a set of patient enrolment packs containing a study information sheet and brief screening questions. Enrolment packs were handed out by receptionists or by the GP to patients with asthma attending appointments and interested in the research. To help identify suitable patients, GPs were offered one-page step-by-step instructions, customized for major Australian practice software packages, for searching practice records by age, prescriptions and condition.

In addition to this resource, we included in the study a number of additional strategies suggested in the literature to encourage GP recruitment of patients:

- Convey the quality and relevance of the research:
  - The research topic was a known priority area for Australian GPs [5,21], our investigator team included a local GP opinion leader and Professor of General Practice and two pulmonologists [5,22], all investigators had extensive experience in primary-care based research, and the logo of the local GP regional organization appeared on GP invitation letters [5,22].
- Provide GP financial reimbursement:
  - $100/patient (see GP recruitment section) [22].
- Provide GP training:
  - As well as receiving training (see RCT Study Design Section), GPs also received continuing professional development points [22] (see RCT Recruitment of GPs section).
- Good communication [7,11], recruitment support and feedback provided to GP by study team [5,22]:
  - GPs received fortnightly telephone support from a co-investigator (JMF) who provided personalised recruitment advice and reminders [22,23], and a monthly study newsletter reporting study recruitment rates and successful recruitment strategies used by participating GPs [22].
- Provide patient incentive:
  - GP-endorsement of the study [24], and one month’s free medication (see RCT Study Design section) [5].

### Methods used in the recruitment barriers and facilitators study

At the end of the trial (mean 17 ± SD4.8 months after the workshop), each GP was asked to complete a self-report questionnaire about barriers/facilitators to patient recruitment, received via fax or email depending on GP preference. GPs who withdrew were sent the questionnaire as soon as possible after withdrawal. A second copy and at least two other reminders were sent to non-responders. The 7-item recruitment questionnaire, based on that of Page et al. [17], consisted of five 7-point Likert scale questions (scored: 1 = strongly disagree; 7 = strongly agree) about the GP’s perceptions of: 1. Intending to approach patients, 2. Not seeing potentially eligible patients, 3. Use of waiting room advertising, 4. Forgetting to approach patients, and 5. Lack of interest from invited patients; and two free text questions. The first question asked about “Anything further the study team could have done to assist in patient recruitment”; the second asked the GP to estimate the total number of patients they recalled inviting into the study. We added two items to the Page questionnaire, to ask if GPs had: 1. screened ineligible patients; and 2. felt study participation took more time than expected. These were scored on the same 7-point Likert scale. The present analysis was planned and questionnaire items decided before commencing recruitment of GPs. This study was approved by the Human Research Ethics Committee, The University of Sydney.

### Data analysis

GP characteristics and questions with Likert scale responses were summarized by frequency distributions and descriptive statistics. Comments provided in open text boxes were independently categorized into themes by JMF and TU; any discrepancies in the themes assigned were resolved by discussion. Comparisons between groups were analyzed by an independent samples *T*-test (two groups) or ANOVA; (more than two groups). Comparisons between the Likert scale responses of recruiter and non-recruiter GPs were analyzed using the Mann–Whitney *U* test. To investigate predictors of patient recruitment by GPs, variables associated with the number of patients enrolled by each GP in univariate analyses (p < 0.1) were entered into a least squares regression model with adjustment for intervention group and GP demographics (age, gender, social disadvantage in GP practice location [low versus high]).

## Results

### Recruitment rates

1662 GPs were sent an invitation letter, and 55 enrolled in and trained for the study; there were no data available regarding the proportion of GPs who responded but failed to meet the inclusion criteria. Recruitment of the original target of 44 GPs took 9 months. After 6 months, they had recruited 119 of the planned 220 patients. In response to emerging low patient recruitment, we recruited and trained a further 11 GPs using the same methods, and extended the patient recruitment phase (for all GPs) to 12 months. Enrolled GPs were demographically similar to the population of Australian GPs with respect to age and gender [25]. Only one GP had a staff member providing recruitment support within the practice.

Forty GPs (age 54.3 ± SD9.0; 40% female) enrolled one or more patients (“Recruiter GPs”) during the study; the 15 GPs who did not enroll patients (“Non-Recruiter GPs”) comprised a higher proportion of females (67%) than the 40 Recruiter GPs (Table 1).

**Table 1:** **GP Practice and GP characteristics in recruiting and non-recruiting GPs**

	**Group 1 GPs who recruited patients N = 40**	Group 2 GPs who did not recruit patients N = 15*
GP age	54.3 ± 9.0	52.3 ± 11.5
% female	40	67
Number of asthma or COPD training events attended by GP in 12 months prior to study enrolment	0.40 ± 0.50	0.60 ± 0.51
% GP practicing in location of social disadvantage^†^	53	60
Number of years practicing as a GP	22.4 ± 11.24	19.3 ± 11.7
Number of months between GP enrolment and first patient enrolled^‡^	2.95 ± 3.96	-----
Total number of GPs working in practice^¶^	4.65 ± 4.6	5.33 ± 2.73
Full time equivalent GPs working in practice^¶^	3.01 ± 3.1	2.75 ± 1.0
% GPs working in practice training registrars^¶^	23	33
% GPs working in practice training medical students^¶^	49	60
Average number of patients seen by GP per week^¶^	165.9 ± 185.9	120.0 ± 69.5
Average number of asthma patients seen by GP per week^¶^	8.56 ± 7.3	12.40 ± 10.8

All variables mean ± SD except where indicated.

*Three GPs continued to study end but did not enroll patients, twelve dropped out or were lost to follow up (sample sizes were too small to make comparison between these sub-groups).

^†^Social disadvantage at GP practice location: ‘Disadvantaged’ SEIFA Quintile ≤3, ‘Advantaged’ SEIFA Quintile: 4–5.

^‡^Range 0–14.8; Median (IQR): 1.25 (0.25, 4.24).

^¶^42 GPs (n = 37 who enrolled patients, n = 5 who did not enroll patients) provided data for these variables.

At completion of patient recruitment, 143 patients had been enrolled at an average of 2.6 ± 2.5 per GP. 15/55 (27%) GPs did not enroll any patients, while 19/55 (35%) reached or exceeded the target of five patients (Table 2). There was no significant difference in patient numbers by trial randomization group (UC: 2.93; PAD: 2.00; IRF: 2.33; IRF + PAD: 3.15; p = 0.641, ANOVA), so data were combined for the remaining analyses. On average, GPs reaching/exceeding the recruitment target enrolled their first patient within 0.9 months, versus 4.8 months in GPs enrolling 1 to 4 patients (p = 0.001, *T*-test).

**Table 2:** **Patient recruitment rate by GPs**

**No. of patients enrolled**	**No of GPs (%)**	**% Female**	**No. of asthma patients seen per week on average by GP** ^*^	**No. months between GP enrolment and first patient enrolled** ^**†**^	**GP recollection of number of patients invited to participate** ^*^	**Proportion of invited patients who were enrolled** ^**‡**^
0	15 (27)	67	12.40 ± 10.78	-----	5.0 ± 5.9	-----
1-4	21 (38)	33	6.65 ± 7.02	4.8 ± 4.7	19.7 ± 25.7	29% ± 34%
5 or more	19 (35)	47	10.9 ± 7.2	0.9 ± 1.2	12.4 ± 6.7	60% ± 30%

Total number of patients enrolled = 143. Mean number of patients enrolled per GP 2.6 ± SD2.5; ^*^Anova, p > 0.149; ^†^Independent samples *T*-test, p = 0.001; ^‡^Anova, p = 0.008. Proportion of invited patients enrolled = No. of patients enrolled/GP recollection of no. patients invited to participate.

Recruitment questionnaires were returned by 93% (37/40) of Recruiter GPs versus 33% (5/15) of Non-recruiter GPs (Table 3). Recruiter GPs reported seeing an average of 8.6 ± 7.3 asthma patients per week, whereas Non-Recruiter GPs reported seeing 12.40 ± 10.8 per week. The number of patients that GPs enrolling 5 or more patients reported having invited was lower than the number reported by GPs who only enrolled 1–4 patients (12.4 ± 6.7 vs 19.7 ± 25.7, Table 2). A non-significant trend was observed by intervention group in the proportion of invited patients who were enrolled (UC: 50%; PAD: 16%; IRF: 21%; and IRF + PAD: 31%; p = 0.071, ANOVA).

**Table 3:** **GPs responses to patient recruitment barrier questionnaire items**

	**Recruiter GPs N=37**	**Non-recruiter GPs N=5**	p-value*
I intended to approach patients	6.1 ± 1.2	5.4 ± 0.9	0.128
I did not see any patients who would have been eligible	2.1 ± 1.6	3.8 ± 2.3	0.073
It was helpful to put a poster or other recruitment material in my practice waiting room	5.3 ± 1.7	3.8 ± 1.3	0.083
I forgot to approach patients with asthma to participate	2.7 ± 1.3	1.8 ± 0.4	0.133
I approached patients with asthma to participate but they were not interested	4.3 ± 1.6	5.4 ± 1.3	0.193
I screened patients with asthma but they were not eligible	5.0 ± 1.3	6.0 ± 1.0	0.128
Participating took more time than I expected	4.7 ± 1.7	5.6 ± 1.9	0.286

All Mean ± SD; All questions included the words “In/for the MICA study” and were scored: 7 = strongly agree, 1 = strongly disagree; *Mann–Whitney *U* test.

### Recruitment barriers

Overall, GPs reported agreement that they intended to approach patients for the study (R: 6.1 ± 1.2; NR: 5.4 ± 0.9), that the asthma patients they had screened were not eligible (R: 5.0 ± 1.3; NR: 6.0 ± 1.0) or not interested (R: 4.3 ± 1.6; NR: 5.4 ± 1.3) and that study participation took more time than expected (R: 4.7 ± 1.7; NR: 5.6 ± 1.9). There were no statistically significant differences in perceived recruitment barriers between GPs who did or did not enroll patients, although differences approached significance (p ≤ 0.083) for GPs’ perceptions of access to eligible patients and usefulness of waiting room advertising (Table 3).

### Free text recruitment barriers and facilitators

A number of barriers were reported by GPs in the free text boxes (Table 4). At the GP-level, some GPs perceived the study to be too intellectual/confronting for patients: “I feel your program is too intellectual for ordinary patients who find instructions too difficult and give up & avoid anything too confronting and being shown up by other parties is unhelpful”, or expressed confusion about recruitment information: “When invited to participate I agreed because I had patients on Symbicort and Seretide. Unfortunately by the time I entered the study only Seretide was an option. I was not prepared to swap patients off Symbicort (my drug of choice)”, or confusion about disease diagnosis and management: “Asthma/COAD i.e. Patient was diagnosed with asthma in the past and later diagnosed with COAD by specialist”.

**Table 4:** **Recruitment barriers and enablers reported by GPs in free text boxes**

**Theme**	**Example**
**Patient level barriers (perceived by GP)**	
Patient unwilling to participate	“Patients who were well controlled on [Diskus] and Turbuhalers were very reluctant to change over to Seretide [Advair] MDI and about 3 patients refused to enter study for this reason.”
Patients unavailable to participate	“Some patients were geographically unavailable: [they worked as fly-in-fly-out employees in mines in Western Australia], some changed address.”
**GP level recruitment barriers**	
Few eligible patients perceived by GP	“I don't think you could have done any more. I guess most of my patients have well-controlled asthma!”
Difficulty prioritizing research due to perceived demands of study or time constraints	“You did a lot to assist recruitment. The excessive amounts of work involved put us off the desire. We then did not give much effort.”
Confusion about recruitment information	“When invited to participate I agreed because I had patients on Symbicort and Seretide. Unfortunately by the time I entered the study only Seretide was an option. I was not prepared to swap patients off Symbicort (my drug of choice).”*
Study thought to be too intellectual or confronting for patients	“I feel your program is too intellectual for ordinary patients who find instructions too difficult and give up & avoid anything too confronting and being shown up by other parties is unhelpful.”
Confusion around disease diagnosis and management	“Asthma/COAD i.e. Patient was diagnosed with asthma in the past and later diagnosed with COAD by specialist.”
**Practice/organization level barriers**	
GP not empowered to recruit within a group practice	“I had a poster in my [office], this did result in some patients volunteering when they read [the] notice (our waiting room is very large and we are prohibited to putting up such posters).”
	“As I am a new GP here I could not/did not try to recruit other doctor's patients into the study. I did not want to take other doctors patients unless they spontaneously moved to see me”.
**Study level barriers**	
Need for more recruitment support	“If a personnel from MICA was sent to help with recruitment that will be a great help for us or for future sites.”
Study materials needed in languages other than English	“I have a lot of patients with limited English, explanation (how to use spacer etc.) in [a language] other than English will help for some.”
Lack of incentive for patient	“It was very hard to convince the patients to participate. [There was] not much incentive.”
**GP level recruitment enablers**	
Good recruitment support	“No! The support and induction processes were excellent.”
Study perceived as beneficial to GPs practice	“MICA study was beneficial personally in learning some new techniques and also had satisfaction [in] that it helped my patients in many ways to improve control and understand their condition.”

*Note - there was no change in the inclusion criteria; the need to switch patients taking another medication at entry was clearly stated during the workshop and in the study materials.

At the practice level, some GPs within group practices reported lack of empowerment when recruiting within a group practice due to practice policy or culture. This included feeling apprehensive about inviting the patients of other GPs or being barred from displaying recruitment posters “I had a poster in my [office], this did result in some patients volunteering when they read [the] notice (our waiting room is very large and we are prohibited to putting up such posters)”.

At the study level, some GPs expressed a need for on-site recruitment support by the study team “If personnel from MICA was sent to help with recruitment that will be a great help for us or for future sites”, while others reported satisfaction with the support provided “No! The support and induction processes were excellent.”

### Variables associated with recruitment outcomes

In backward linear regression, after adjustment for intervention group and GP demographics (age, gender, social disadvantage in GP practice location), lower recruitment was associated with a longer time to first patient enrolled (B-Coefficient −0.482, 95% CI: −0.41 to −0.11), GP-perceived poor access to eligible patients (B-Coefficient −0.273, 95% CI: −7.59 to 0.01) and GP working in a practice training medical students (B-Coefficient −0.252, 95% CI: −2.28 to 0.07). The explained variance of the model was 48%. One variable was associated univariately with higher recruitment (stronger GP reporting of their intention to approach patients, Spearman's rho 0.265, p = 0.095) but this was not associated with recruitment in the final model.

## Discussion

In this primary care-based professional-cluster RCT, although we planned a modest target of 5 patients per GP, utilized a range of strategies thought to enhance recruitment and extended the recruitment period, GPs recruited an average of only 2.6 patients each. At study end, although the RCT itself was sufficiently powered for its primary outcome, only one third (35%) of GPs had met the recruitment target and 27% had failed to enroll a single patient. In this follow-up survey, GPs provided considerable insight into barriers and facilitators that had affected their success at recruiting patients for the study.

Recent qualitative research [4] and recent systematic reviews about recruitment by clinicians for RCTs in general [26] and in primary care [5] have highlighted the difficulty of fitting research recruitment into daily practice. Research specifically investigating patient recruitment by GPs suggests facilitators may include relevant research with perceived benefit or incentive for patients and GPs [6-10], good communication between researchers and GPs [7,11] and minimising the research responsibilities of GPs. Possible barriers include patients’ unwillingness to participate [9], competing demands on GPs’ time [10,12,13] and a lack of research interest in individual GPs or their practice [8,9,14]. However, much of this research is based on inferences from researchers’ rather than GPs’ experiences, and studies often involved pharmacological interventions or interventions with relatively low GP burden.

By contrast, primary care professional-cluster RCTs, in which the GP is not only required to recruit participants from amongst their patients, but also to deliver the (non-pharmacological) intervention in which they have been trained, provides additional challenges. We are aware of only one previous primary care professional-cluster RCT study in which recruitment barriers were assessed [17]; it found barriers to recruitment such as GPs forgetting to recruit patients, GP time constraints and lack of patient interest or incentive for participation. The information provided by this previous study is somewhat different from the present study because it required less involvement from GPs (the study aimed to improve GP adherence to clinical practice guidelines for acute low-back pain specifically by reducing patient referral for x-ray), and recruitment of patients was abandoned due to very poor enrolment rates, although the investigators introduced clinical vignettes and recorded process measures to continue their research [27].

In the present study in which GPs recruited patients and delivered the intervention, recruitment of patients was impeded by GPs perceiving that they had poor access to eligible patients, and by a delay in the time it took GPs to enroll their first patient. While there may have been real differences between GPs in their access to eligible patients, such as between-practice differences in patient populations, all GPs had at least 12 months for recruitment which suggests that recruitment delays were also influenced by GP factors. We do not know, for example, if participating GPs had had previous research experience. Recruitment requires skills which GPs may lack opportunity to develop, including confident explanation of study requirements and accepting patient choice to not participate [14]. Initial failure to enroll patients could negatively impact GPs’ perceptions of study feasibility and motivation, and thus slow or halt recruitment attempts [14]. Recruitment appeared lower in busier practices, as indicated by low-recruiting GPs seeing a higher number of asthma patients per week, and being involved in training of medical students; training obligations may have reduced available time for recruitment.

Comments from GPs suggest a complex array of barriers and facilitators at each level: the GP, the Practice setting, the Patient, and the Study. At the GP-level, the study was considered by some GPs to be too intellectual or confronting for their patients, perhaps relating to the focus on adherence issues. Patient ineligibility may have been appraised subjectively by some GPs rather than objectively based on formal study inclusion criteria. The deliberate exclusion of patients perceived by the GP to be ‘vulnerable’ has been described in other primary care studies as ‘gatekeeping’ [12,14]; restricting the opportunity for some patients to hear about a study and consider whether they wish to participate has important consequences for the representativeness of trial results.

As previously observed [5,10,14], some GPs in the present study reported difficulty prioritizing research due to perceived study demands or time constraints. All three GPs reporting this were randomized to deliver PAD. Although this was expected to add about 15 extra minutes to an asthma consultation, the GPs and their patients would have had access to reimbursement for this extra time through the Australian Medical Benefits Schedule. There was no difference in the mean number of patients recruited by randomization group (p = 0.641), and many PAD GPs met the recruitment target. This suggests that individual GPs’ attitudes towards research could be as important for recruitment as minimizing the research burden.

At the practice level, some GPs working in group practices reported disempowerment around research. They described prohibition of study advertising in the waiting room and feeling apprehensive about inviting the patients of other GPs within the practice. Organizational barriers to conducting research were reported in another primary care-based RCT, where some GPs not involved in the study obstructed colleagues’ participation directly by objecting, or indirectly through practice systems which restricted access to patient data by individual GPs [7].

At the study level, some GPs reported the need for more recruitment support, such as provision of study staff within their practice to assist with recruitment. In contrast other GPs reported good recruitment support which facilitated recruitment. Perceived poor recruitment support is a barrier described in other studies [5,14]. However, a key strength of real-world primary care based research is generalizability of the results to other similar populations.

At the patient level, one barrier was the need for some patients to change their inhaler type. This was because, at the time the study was conducted, objective monitoring of controller medication use was only possible for pressurized metered dose inhalers. Some GPs reported that patients were reluctant to switch inhaler and therefore did not participate. Although guidance was provided, the need to have this conversation with some patients may have increased the perceived complexity of the study, which despite being a known barrier to recruitment [10,28] could not be avoided for reasons of scientific rigor.

Limitations of our research include that GP self-report data were retrospective and returned to a research team known to the GP which may have biased GP responses. We were unable to obtain reliable prospective data on the number of patients invited as GPs failed to retain screening documents despite a request to do so. Future studies should aim to collect data prospectively and anonymously. Our GP sample size for this analysis was opportunistic, comprising all GPs recruited for the cluster RCT [18], but likely under-powered for the present comparisons between recruiting and non-recruiting GPs. The low survey response rate among non-recruiting GPs implies that our results are more representative of recruitment barriers associated with recruitment continuance than recruitment initiation; barriers associated with first patient enrolment, such as difficulty navigating new research procedures or explaining the study to patients for the first time [14,28], may be somewhat under-represented in the study.

Although the low response rate of GPs enrolling into the study (3.3%) was not atypical of Australian primary care-based studies [29,30], the GPs who enrolled in this study may have been biased toward those with an interest in asthma or in research participation. The limited representativeness of RCT samples to the general population is an ongoing and common problem [31] and our results, like those of other RCTs, may not be generalizable to harder to reach populations. Our results may also have limited generalizability to professional-cluster RCTs carried out in countries with different health systems, to more or less burdensome or sensitive interventions and for different medical conditions. However as described above, this is the first study investigating barriers and facilitators to patient recruitment amongst GPs participating in a completed professional-cluster RCT and as such, provides valuable information on the issues faced by GPs in these complex but crucial studies.

Our findings suggest strategies that may be useful to enhance recruitment in future cluster randomized primary care studies. Provision of brief GP training on patient recruitment skills may be useful, although gaining direct access to GPs can be challenging [7], and hands-on experience is generally recommended over formal recruitment training [4]. More intensive recruitment support, for example with on-site study staff, could enhance enrolment rates particularly if delivered early in the recruitment phase to capitalize on the momentum from training, but care should be taken to avoid interference with usual care. It may be helpful to identify GPs who are most likely to need support, for example those in busy practices. Given comments from several GPs about real or perceived limitations to recruitment within group practices, randomization by practice rather than by individual GP could encourage a more collegial approach to recruitment. However, a cultural change toward research may be needed and may require specific intervention such as the “Research Ready Accreditation” scheme for general practices in the UK [32]. A longer-term strategy could be to provide medical students with education about primary care research and related ethical concepts, such as informed consent and withdrawal rights. A recent systematic review of interventions to improve clinicians’ recruitment of patients to randomized controlled trials (although not limited to primary care research) came to a similar conclusion [26]. Finally, minimization of GP burden in both recruitment and study procedures should continue to be a priority, and where time-consuming tasks cannot be avoided, lower patient recruitment rates may need to be incorporated into sample size calculations.

## Conclusions

In a professional-cluster RCT requiring significant commitment from GPs, only one-third of GPs reached the (modest) planned patient recruitment target. Patient recruitment was influenced by a number of barriers and facilitators at the level of the GP, practice setting, patient and study. Although some barriers in professional-cluster RCTs may be difficult to address such as the cultivation of a research culture in general practice, others appear modifiable, for example through provision of training on recruitment skills and expectations. Barrier modification will need to strike a balance between scientific rigor and feasible processes for primary care settings. We strongly recommend that future researchers include evaluations of the recruitment strategies used in their studies, to build a stronger evidence base around effective approaches and to optimize the ethical undertaking and successful completion of future primary-care based professional-cluster RCTs.
